# Endoloop Versus Metallic Clip for Stump Closure in Laparoscopic Appendectomy: A Systematic Review and Meta-Analysis

**DOI:** 10.7759/cureus.77471

**Published:** 2025-01-15

**Authors:** Diogo S Almeida, Luis Henrique A Medina, Elaine R Coelho, Eduardo R Braga, Vitor Luiz V Martinez, Gilson T Oliveira, Cecília G Soares, Guilherme A Figueiredo, Luiza A Baz

**Affiliations:** 1 General Surgery, Bahiana School of Medicine and Public Health, Salvador, BRA; 2 General Surgery, Zarns Health Education Institute, Salvador, BRA; 3 Digestive System Surgery, Roberto Santos General Hospital, Salvador, BRA; 4 General Surgery, Salvador University (UNIFACS), Salvador, BRA

**Keywords:** appendectomy, appendectomy techniques, appendiceal stump, appendicular stump closure, clip, endoloop, laparoscopy, metallic, stump

## Abstract

Appendectomy is the treatment of choice for most cases of acute appendicitis, and, considering that the availability of technical variables for closing the appendicular stump raises questions for surgeons, analyzing which closure method provides the best results is necessary. This study aims to compare the variables of operating time, readmission rate, reoperation, and surgical wound infection regarding the endoloop and metallic clip techniques for closing the appendicular stump in laparoscopic appendectomy. Only randomized clinical trials that compared metallic clip and endoloop appendicular stump closing in laparoscopic appendectomy were used. Of these, those that did not fit the PICOT (Patient, Intervention, Comparison, Outcome, and Time) question and inclusion criteria were excluded, with five articles being included for the analysis phase. Five randomized clinical trials were included in the statistical analysis, totaling a population of 344 patients, with 181 patients in the metal clip group. It was observed that the operation time was significantly shorter in the metal clip group (STD -0.81, 95% CI: -1.13, -0.49, p < 0.00001, I² = 50%). To the extent that the reoperation rate (RR 1.27, 95% CI: 0.20, 7.88, p = 0.80, I² = 0%), the readmission rate (RR 0.93, 95% CI: 0.29, 2.91, p = 0.89, I² = 0%), and the surgical wound infection rate (RR 0.87, 95% CI: 0.24, 3.13, p = 0.83, I² = 0%) were statistically similar between the two groups. The findings of this study demonstrate that the closure of the appendicular stump using metal clips in laparoscopic appendectomy is faster, with similar rates of readmission, reoperation, and surgical wound infection between the two groups.

## Introduction and background

Acute appendicitis is the most frequent cause of acute inflammatory abdomen [1], with an incidence of 1 in every 1000 people per year [2]. It was first described by Fitz in 1886 and is also the most common cause of acute abdominal pain [3]. This disease involves obstruction of the appendiceal lumen, which can be due to a foreign body, fecolith, or inflammatory process, the latter being its main pathophysiological agent [1].

Furthermore, the lifetime risk of acute appendicitis is slightly higher in men than in women (8.6% versus 6.7%), but women have a higher lifetime risk of undergoing appendectomy (23.1% versus 12.0%). Moreover, adolescent girls (ages 12-16) are the group at greatest risk for appendectomy [2].

Clinically, it is characterized by pain, initially located in the epigastric and periumbilical regions, and later shifting to the right iliac fossa (RIF). It has a continuous nature, worsens with movement, and might be accompanied by nausea, vomiting, fever, and shivering. The general and hemodynamic conditions are usually preserved. On physical examination, the patient is commonly noted to have reduced mobility, an antalgic attitude, peritoneal irritation, and reduced bowel sounds. The diagnosis is supported by laboratory tests, such as a hemogram, and imaging methods, like ultrasound and tomography [1].

Regarding treatment, nearly all patients with acute appendicitis undergo appendectomy as the ideal therapeutic method, whether by open or laparoscopic technique. The latter was first described in 1983 and has been preferred in most cases nowadays [1,3]. Additionally, a significant variation among surgeons exists in the method of appendiceal stump closure, either with metallic clips or an endoloop. The choice remains subjective and generates some uncertainty about which technique offers greater safety and fewer adverse events. Therefore, this review aims to highlight which method of appendiceal stump closure has fewer adverse events and better postoperative recovery.

Previous studies have proposed that, for perioperative complications, there was no significant difference between the metallic clip and endoloop. In addition, no differences were noted in the length of hospital stay. However, a significant reduction in operative time was observed with endoloop as opposed to the metallic clip [3-6]. When analyzed alone or compared with other techniques besides the metallic clip, the endoloop proves to be a great option for closing the appendicular stump, including in children, with lower costs and similar complication rates to other techniques. Finally, it is proving to be a great option to help solve a problem that causes over 250,000 hospitalizations each year in the United States [7-10].

## Review

Materials and methods

Literature Search

Articles were selected from the electronic bibliographic databases Embase and PubMed, covering the years from 2014 to 2024, comparing the use of endoloop versus metallic clips in laparoscopic appendectomy. The following search terms were used in the search algorithm: "laparoscopic appendectomy" OR "appendectomy" AND "stump" OR "stump closure" OR "stump closure techniques." The study was registered in the International Prospective Register of Systematic Reviews (PROSPERO) (CRD42024566095).

Study Selection

Only randomized clinical trials comparing metallic clips and endoloop for laparoscopic appendectomy were included in the study. Study identification and subsequent data extraction were independently performed by four researchers, with conflicts resolved by consensus. The Rayyan software (Qatar Computing Research Institute, Ar-Rayyan, Qatar) [11] was used for the initial screening of articles, where four researchers independently and blindly selected 18 out of 584 articles. In the PICOT (Patient, Intervention, Comparison, Outcome, and Time) question: the population was patients undergoing laparoscopic appendectomy, the intervention was Hem-o-lok stump closure and metallic clips stump closure, the control group was endoloop stump closure, and the outcomes were operative time, costs, perioperative time, morbidity, and complications. Of the 18 selected articles, those that did not meet the PICOT question and inclusion criteria were excluded, resulting in five articles being included in the analysis phase. No language restrictions were imposed.

Data Extraction

Study details, patient characteristics, and outcome data were independently extracted by four reviewers. The primary outcome was operative time. Secondary outcomes included length of hospital stay, reoperation rate, surgical wound infection, and readmission rate. The following data were obtained from the included studies: first author, year of publication, study design, number of patients in each group, age, and gender.

Quality Assessment

This systematic review and meta-analysis was conducted according to the Preferred Reporting Items for Systematic Reviews and Meta-Analyses (PRISMA) guidelines [12]. The risk of bias was assessed using the Revised Cochrane risk-of-bias tool for randomized trials (RoB 2) [13], and studies with low methodological quality or duplicates were excluded. Any potential conflicts were discussed with a third reviewer.

Statistical Analysis

Categorical outcomes (reoperation rate, readmission rate, and wound infection rate) were expressed using a risk ratio with 95% CIs. Continuous variables (operative time) were analyzed using the standardized mean difference. Data originally reported as median and interquartile range were converted to mean and standard deviation.

Studies that did not explicitly report key information required for analyzing these outcomes in their tables were handled by actively searching the results presented in the text.

The DerSimonian and Laird random-effects model was applied. Heterogeneity was assessed using the I² statistic, with the following thresholds for interpretation: I² less than 25% (low heterogeneity), I²: 25%-50% (moderate heterogeneity), I²: 50%-75% (substantial heterogeneity), and I² greater than 75% (high heterogeneity). Statistical analysis was performed using Review Manager software, version 5.4.1 [14].

Results

Study Selection

**Figure 1:**
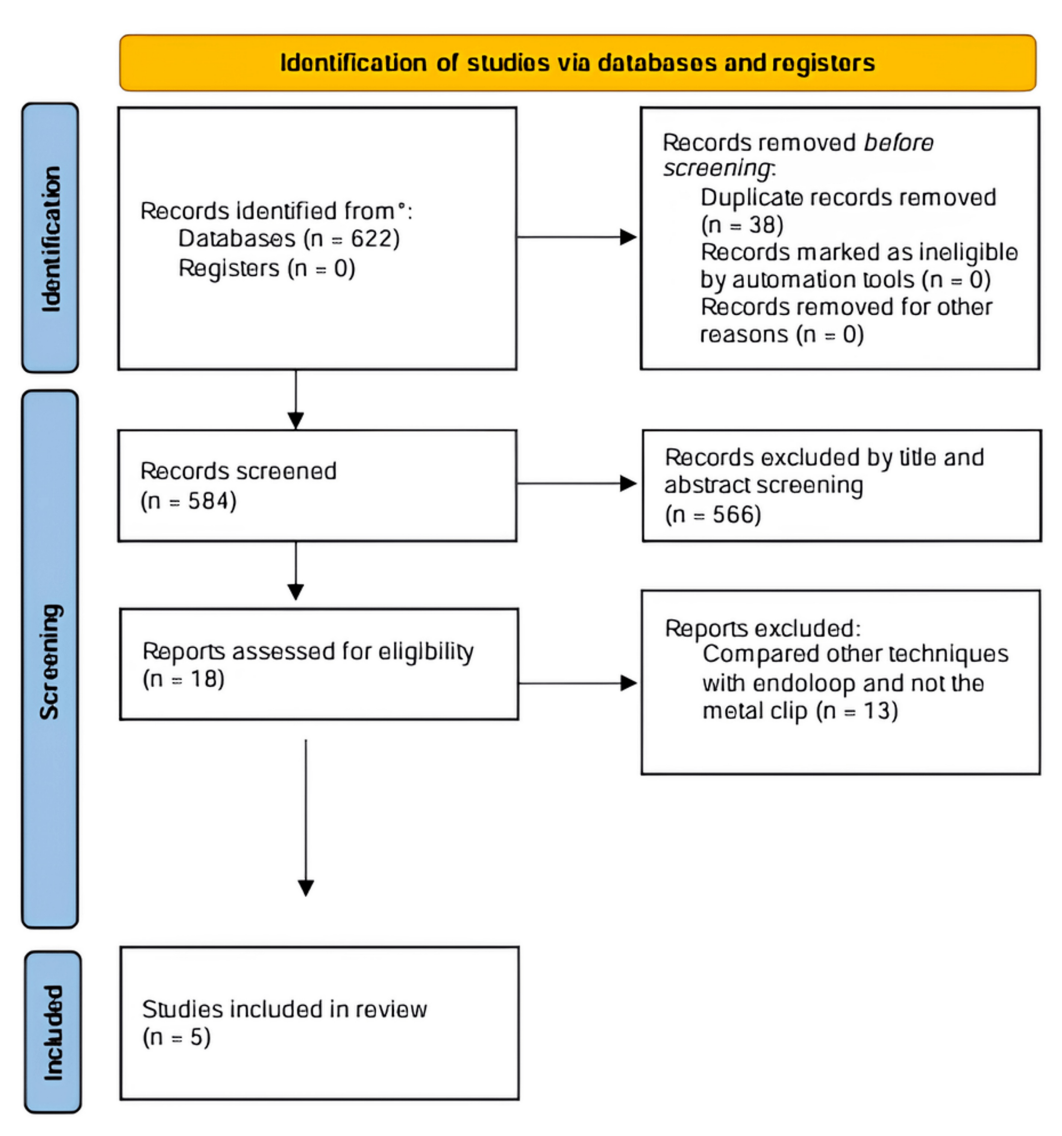
PRISMA 2020 flow diagram PRISMA, Preferred Reporting Items for Systematic Reviews and Meta-Analyses

Through a preliminary database search, 601 studies were obtained. After removing duplicates with the help of the Rayyan program, 539 titles and abstracts were screened, leaving 18 studies for full-text evaluation. As shown in the flowchart (Figure 1), only five manuscripts fulfilled the eligibility criteria for this meta-analysis, all of which were randomized controlled trials.

Study Characteristics

**Figure 2:**
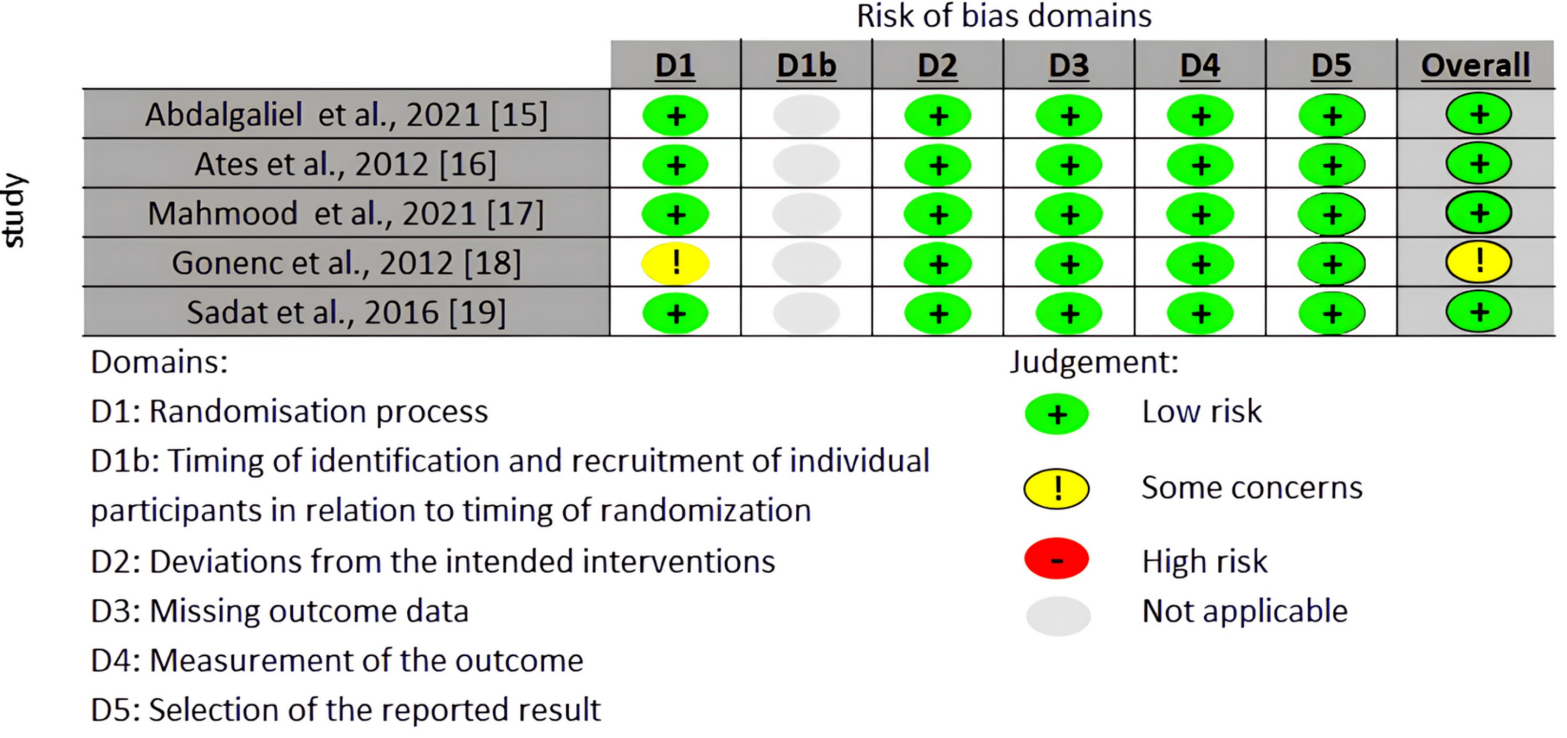
Risk of bias (RoB 2) summary for randomized studies

A total of five studies were included, comprising 344 individuals. The main characteristics of all included studies are shown in Table 1, while the risk of bias is shown in Figure 2.

**Table 1:** Main characteristics of included studies RCT, randomized clinical trial

Study	Type of study	Patients, no.	Female, no. (%)	Age (y), endoloop	Age (y), metal clips		Main findings	
Abdalgaliel et al. (2021) [15]	RCT	40	55	NA	NA		The incidence of complications was higher in the clipping group but the operative duration was lower than the endoloop group.	
Ates et al. (2012) [16]	RCT	61	59	29.35 ± 8.2	28.23 ± 11.1		The mean operative time for the endoclip group (41.27± 12.2 min) was shorter than that for the knot-tying group.	
Mahmood et al. (2021) [17]	RCT	68	17	24 ± 7.78	23.9 ± 7.3		Metal clips group had a shorter operative time, but more postoperative complications.	
Gonenc et al. (2012) [18]	RCT	117	47.8	27.40 ± 11.48	26.76 ± 13.25		The endoloop group had more postoperative complications and a higher operative time.	
Sadat-Safavi et al. (2016) [19]	RCT	76	50	24.26 ± 5.9	22 ± 3.6		The duration of surgery in endoclip groups was lower.	

Outcomes

It was observed that operation time was significantly shorter in the metallic clip group (standardized mean difference = -0.81, 95% CI: -1.13, -0.49, p < 0.00001, I² = 50%). Additionally, reoperation rate (RR = 1.27, 95% CI: 0.20, 7.88, p = 0.80, I² = 0%), readmission rate (RR = 0.93, 95% CI: 0.29, 2.91, p = 0.89, I² = 0%), and surgical wound infection rate (RR = 0.87, 95% CI: 0.24, 3.13, p = 0.83, I² = 0%) were statistically similar between the two groups.

Meta-Analysis

**Figure 3:**
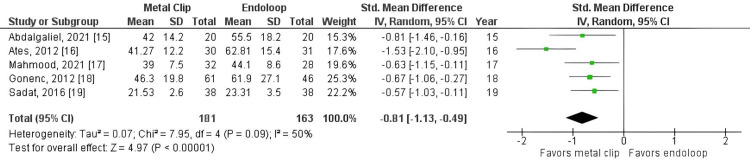
Metal clip x endoloop comparing operative time

**Figure 4:**
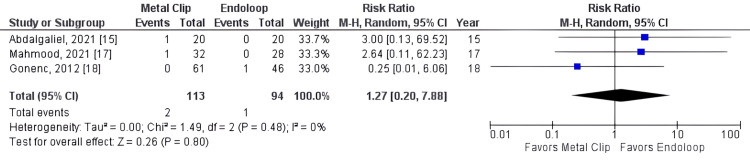
Metal clip x endoloop comparing reoperation rate

**Figure 5:**
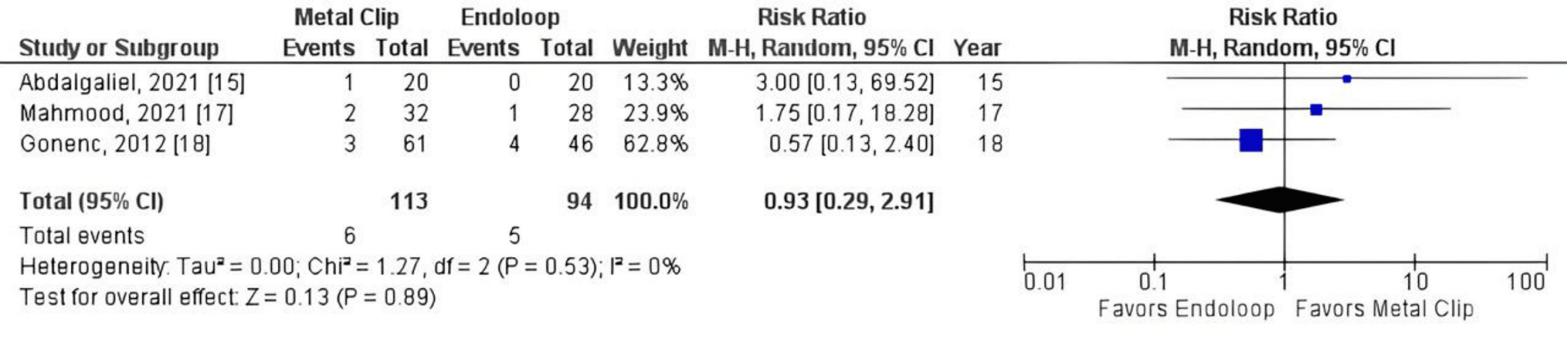
Metal clip x endoloop comparing readmission rate

**Figure 6:**
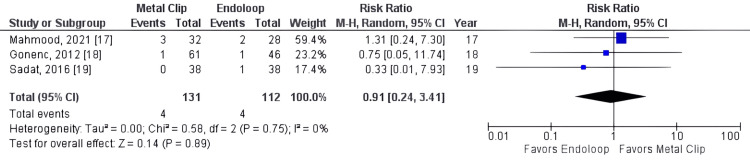
Metal clip x endoloop comparing surgical wound infection

All studies reported operative time as an outcome (studies 15, 16, 17, 18, 19). The total combined effect size (standardized mean difference) was -0.81, 95% CI: -1.13 to -0.49. The Z-score for the overall effect was 4.97, with a p-value < 0.00001, indicating a highly significant overall effect, as shown in Figure 3. However, there was no statistically significant difference between groups for reoperation (RR = 1.27, 95% CI: 0.20, 7.88, p = 0.80, I² = 0%), readmission rate (RR = 0.93, 95% CI: 0.29, 2.91, p = 0.89, I² = 0%), and surgical wound infection rate (RR = 0.93, 95% CI: 0.29, 2.91, p = 0.89, I² = 0%), as shown in Figures 4-6.

Discussion

Our analysis demonstrates that patients who underwent appendectomy treated with metal clips had a significantly shorter operative time compared with those treated with the endoloop technique, while readmission and reoperation rates, along with wound infections, were similar between the two groups.

Clinical Outcomes

Analyzing operative time, all studies individually showed the metal clip technique as significantly faster when compared with the endoloop group [15-19]. Three studies brought the reoperation rate as an outcome [15,17,18]. Abdalgaliel et al. [15] and Mahmood et al. [17] both had one reoperation event in the metal group, while Gonenc et al. [18] had one reoperation event in the endoloop group. Numerically, more reoperation events were found in the metal clip group, but they were not statistically significant.

The same studies also brought readmission rates as an outcome [15,17,18]. Gonenc et al. [18] had three readmission events in the metal group and four in the endoloop group, being the study with the biggest population that analyzed this outcome. However, Mahmood et al. [17] had two readmission events in the metal group and one in the endoloop group. In addition, Abdalgaliel et al. [15] had one readmission event in the metal group. As observed in the reoperation rate, numerically, more readmission events were found in the metal group, but they were not statistically significant.

The last outcome analyzed was surgical wound infection. Three studies included it in their analyses [16-18]. Events were equally distributed between the metal and endoloop groups, with four events each, but were not statistically significant.

Comparison With Previous Studies

A previous meta-analysis compared endoclips versus endoloop in stump closure. The results found were similar to those in this study: endoclips are faster, with similar complication rates. However, this previous study compared not only metal clips but also other types of endoclips. In addition, non-randomized and observational studies were included in their analysis [20]. Other meta-analyses found the metal clip technique to be associated with a higher number of surgical wound infections and a lower number of organ space infections, both statistically significant. Nevertheless, this study used indirect comparison to arrive at these results [21].

There is still no consensus in the literature regarding the best technique; however, some studies found similar results regarding the outcomes analyzed, including the complication rate and readmission rate [4,5,9,15,17,18]. Furthermore, in relation to operative time and costs, some studies showed a significant difference in techniques, with the endoloop being the lowest cost and the metal clip being the fastest [3,4,6,7]. However, one older study proposed that the metal clip would be cheaper than the endoloop, costing 7 dollars, while the endoloop would cost 50 [6]. Another study showed that the endoloop is a safe and effective technique for closing the appendicular stump in children, including in cases of complicated appendicitis [8].

Limitations of the study

The limitations of this study include the limited number of available articles. Only five randomized clinical trials met the inclusion criteria and were limited by the small sample size. Furthermore, we observed significant heterogeneity (>25%) among studies when analyzing operative time. Lastly, we were unable to perform subgroup and sensitivity analyses due to the small number of included studies.

## Conclusions

In conclusion, the use of metal clips and endoloop for appendicular stump closure demonstrated comparable outcomes in terms of readmission rates, reoperation rates, and wound infections. However, metal clip closure was significantly faster, leading to shorter surgical times. The analysis was limited by the inclusion of only five randomized clinical trials with small sample sizes. Further, well-designed randomized clinical trials are needed to provide more definitive insights, particularly regarding the cost-effectiveness of these techniques.
